# Wharton’s Jelly–Mesenchymal Stem Cell–Engineered Conduit for Pulmonary Artery Reconstruction in Growing Piglets

**DOI:** 10.1016/j.jacbts.2021.11.013

**Published:** 2022-02-23

**Authors:** Filippo Rapetto, Dominga Iacobazzi, Srinivas A. Narayan, Katie Skeffington, Tasneem Salih, Shahd Mostafa, Valeria V. Alvino, Adrian Upex, Paolo Madeddu, Mohamed T. Ghorbel, Massimo Caputo

**Affiliations:** a Department of Cardiac Surgery, Bristol Royal Hospital for Children, Bristol, United Kingdom; b Translational Health Sciences, University of Bristol, Bristol, United Kingdom; c Department of Paediatric Cardiology, Bristol Royal Hospital for Children, Bristol, United Kingdom; d Department of Anaesthesia, Bristol Royal Hospital for Children, Bristol, United Kingdom

**Keywords:** growing swine model, right ventricular outflow tract reconstruction, small intestinal submucosa, tissue engineering, CMR, cardiovascular magnetic resonance, FISH, fluorescent in situ hybridization, MPA, main pulmonary artery, RVOT, right ventricular outflow tract, SIS, small intestinal submucosa, SMA, smooth muscle actin, TTE, transthoracic echocardiography, WJ-MSC, Wharton’s Jelly–mesenchymal stem cells

## Abstract

Surgical treatment of congenital heart defects affecting the right ventricular outflow tract often requires complex reconstruction and multiple reoperations. With a randomized controlled trial, we compared a novel tissue-engineered small intestine submucosa–based graft for pulmonary artery reconstruction (seeded with mesenchymal stem cells derived from Wharton’s Jelly) with conventional small intestine submucosa in growing piglets. Six months after implantation, seeded grafts showed integration with host tissues at cellular level and exhibited growth potential on transthoracic echocardiography and cardiovascular magnetic resonance. Our seeded graft is a promising biomaterial for pulmonary artery reconstruction in pediatric patients with right ventricular outflow tract abnormalities.

## Highlights

• Tissue engineering technologies are a promising tool to overcome the limitations of currently available materials for RVOT surgery in the pediatric population.
• Porcine SIS seeded with allogeneic WJ-MSCs with our technique is a safe and reliable biomaterial for RVOT surgical reconstruction on a growing swine model.
• Cell-seeded porcine SIS shows better integration with the host compared to porcine SIS alone, both in terms of histological/microscopic findings and in terms of clinically measurable radiological outcomes, as assessed by echocardiography and cardiovascular magnetic resonance.

## Visual Abstract

**Figure:**
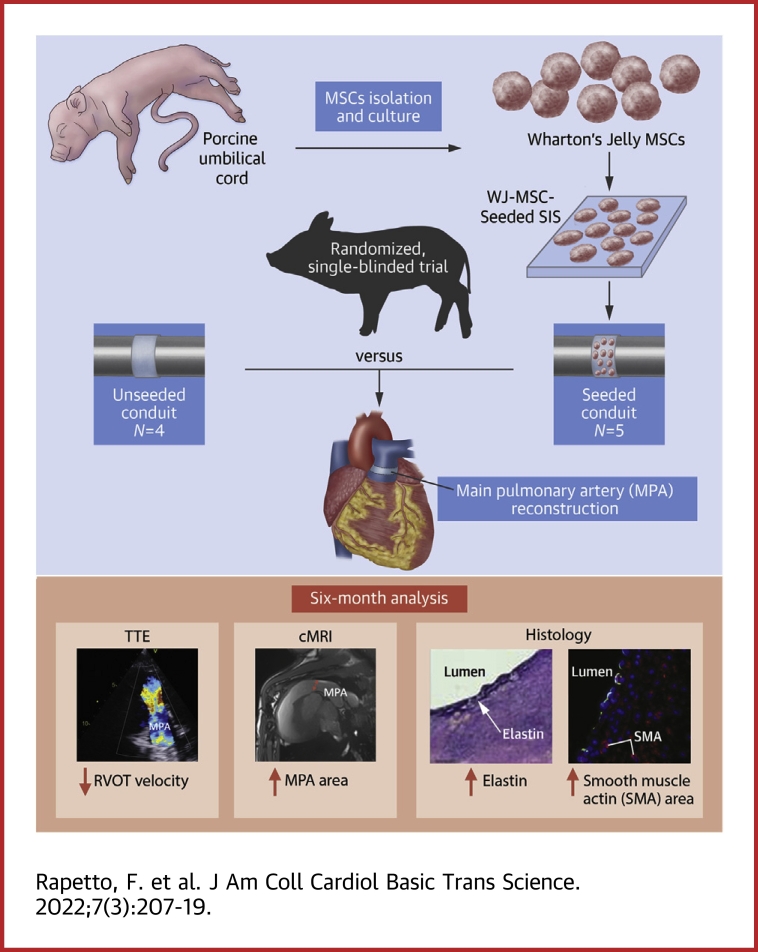

Treatment of congenital heart defects in children requiring right ventricular outflow tract (RVOT) reconstruction, such as tetralogy of Fallot with pulmonary stenosis or pulmonary atresia and truncus arteriosus, often requires implantation of a prosthetic right ventricle to pulmonary artery conduit, and typically involves multiple open-heart surgeries because of intrinsic limitations of all the existing graft materials.^1^, ^2^, ^3^, ^4^, ^5^ Although homografts have been the most used conduits for RVOT reconstruction to date, they have less than ideal performance in children, especially in neonates and infants.^6^^,^^7^ Along with poor performance, limited homograft availability has prompted a search for alternative conduits, including bovine jugular vein (Contegra, Medtronic), or Dacron (polyethylene terephthalate) conduits. All these conduits do not remodel or grow, requiring replacement in significant proportions of patients (30% to 40%) in the first few years of life caused by both somatic outgrowth and structural degeneration.^6^, ^7^, ^8^, ^9^, ^10^

Recellularizing acellular graft materials and generating a conduit with growth potential through tissue engineering is a promising approach to this clinical problem.^5^^,^^11^, ^12^, ^13^, ^14^, ^15^

Our group has published a reproducible technique to harvest and expand mesenchymal stem cells from Wharton’s Jelly–derived mesenchymal stem cells (WJ-MSCs), and to implant them onto commercially available patches of porcine small intestinal submucosa (SIS) (ProxiCor [previously CorMatrix] Inc). We and others have found that WJ-MSCs are characterized by easy accessibility, extended plasticity, self-renewal capacity, long-term expansion, as well as immunoprivileged properties.^16^, ^17^, ^18^, ^19^

Moreover, we have developed a growing swine model to test our tissue-engineered biomaterial: pilot data validated our preclinical model and demonstrated the safety of the engineered WJ-MSC SIS graft for reconstruction of the main pulmonary artery (MPA).^13^ This study specifically focused on MPA reconstruction and it is the first step toward the development of a novel valved conduit. We compared our tissue-engineered vascular graft to commercially available SIS (ProxiCor) through a randomized controlled trial in piglets.

## Methods

### Animals

Porcine umbilical cord samples were collected within 24 hours after birth from Landrace female piglets (average weight: 1.5 kg) sacrificed using Schedule 1, following the guidelines of the UK Home Office. Four-week-old female Landrace pigs were used for in vivo graft implantation studies under UK Home Office ethical approval PF6E6335D. Animals were treated in accordance with the “Guide for the Care and Use of Laboratory Animals” published by the National Institutes of Health in 1996 and conforming to the “Animals (Scientific Procedures) Act” published in 1986.

### Porcine WJ-MSCs culture and graft cellularization

WJ-MSCs were isolated from umbilical cord of newborn female piglets by mechanic dissociation, as previously described.^19^ The isolated cells were fed with fresh medium every 3 days and expanded until passages 3 to 5. Fluorescence-activated cell sorting analysis was used to determine cell surface marker expression.^12^^,^^19^

Expanded WJ-MSCs (passage 2 to passage 5) were seeded onto decellularized porcine SIS at a density of 2.5 × 10^5^/cm^2^ and cultured until graft maturation according to the protocol previously optimized by our group.^11^, ^12^^,^^19^

### In vivo experiments

**Figure 1:**
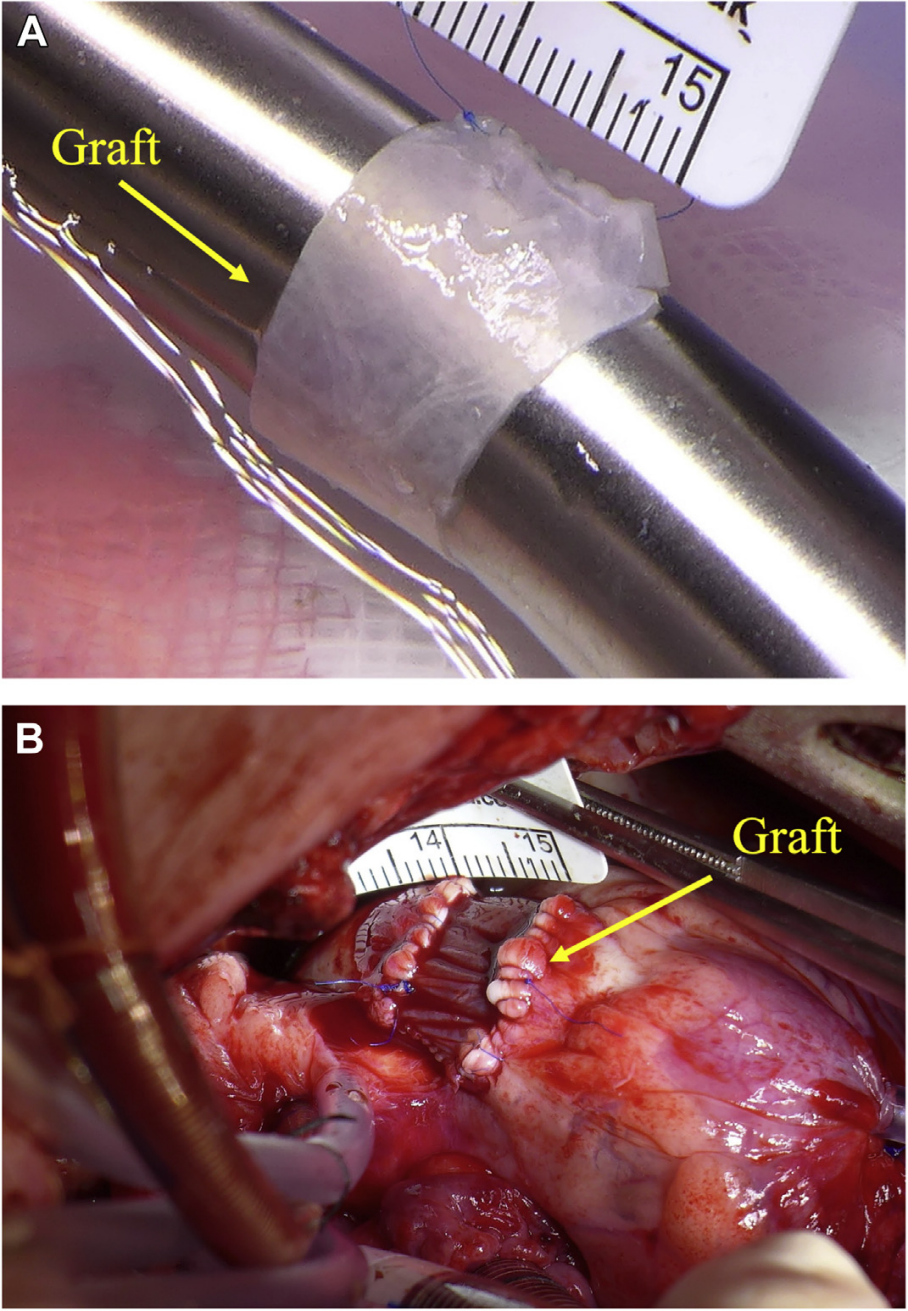
Graft Preparation and in Vivo Implantation **(A)** Circumferential graft created from patch of small intestinal submucosa (running longitudinal suture visible). **(B)** Graft after implantation on main pulmonary artery.

A total of 11 4-week-old healthy Landrace female piglets (mean weight: 20.5 ± 4.1 kg) were used in this study (9 for comparison between seeded and unseeded grafts, and 2 to define the fate of the WJ-MSCs). On the day of surgery, all animals were premedicated with ketamine 10 μg/kg and/or dexmedetomidine 15 μg/kg injected intramuscularly; general anesthesia was induced with intravenous propofol and maintained with isoflurane in oxygen. A continuous intravenous infusion of pancuronium bromide (0.1 mg/kg per hour) was used to achieve neuromuscular blockade. Immediately before starting the surgical procedure, transthoracic echocardiography (TTE) was performed. Following this, a circumferential graft was created from a rectangular SIS patch (length: 10-12 mm, circumference: MPA diameter as measured by TTE, multiplied by 3) as shown in Figure 1A. Four animals were randomized to receive a graft made from commercially available SIS, whereas 5 animals received a WJ-MSC–seeded graft; the surgeon was blinded to the graft seeding.

**Figure 2:**
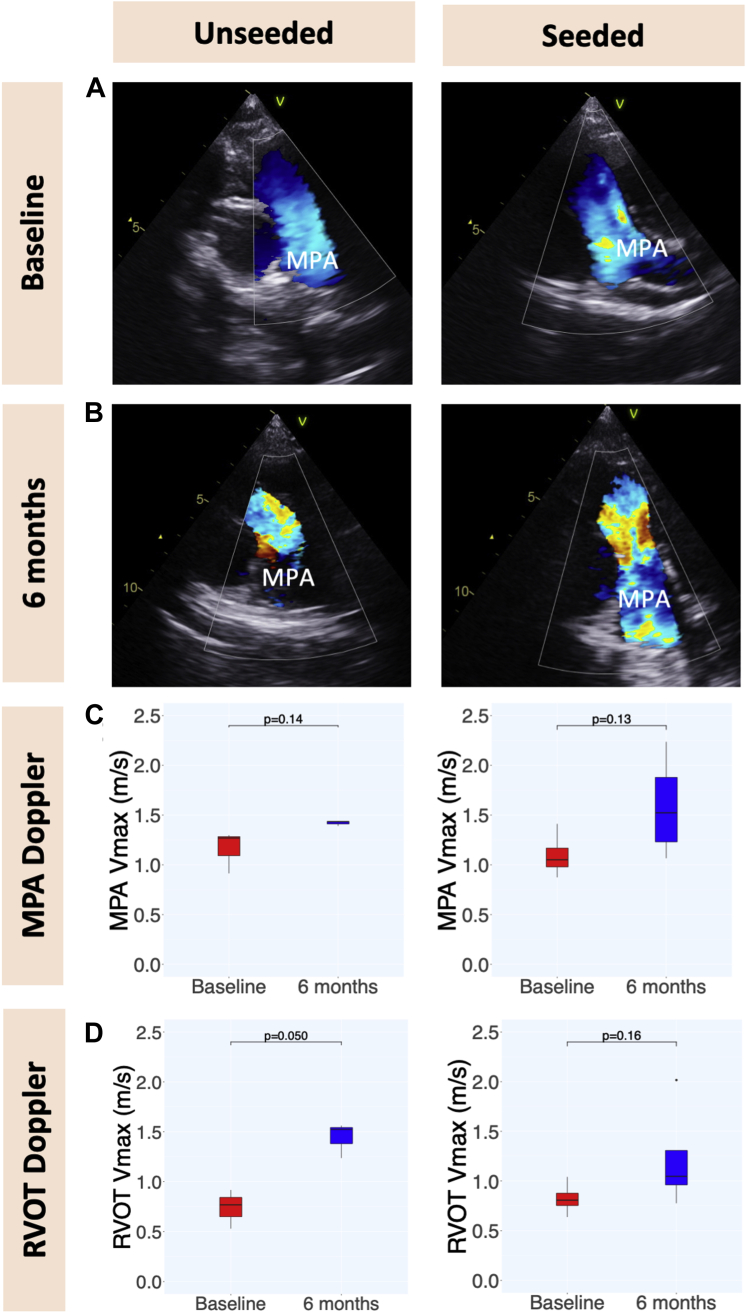
Doppler Analysis **(A,B)** Color Doppler images of the main pulmonary artery at baseline and 6 months following graft implantation in seeded and unseeded animals. **Box plots** summarizing Doppler measurements on MPA **(C)** and RVOT **(D)** at baseline and at 6-month follow-up, in unseeded and seeded animals (n = 4, ± SD). MPA = main pulmonary artery; RVOT = right ventricular outflow tract.

**Figure 3:**
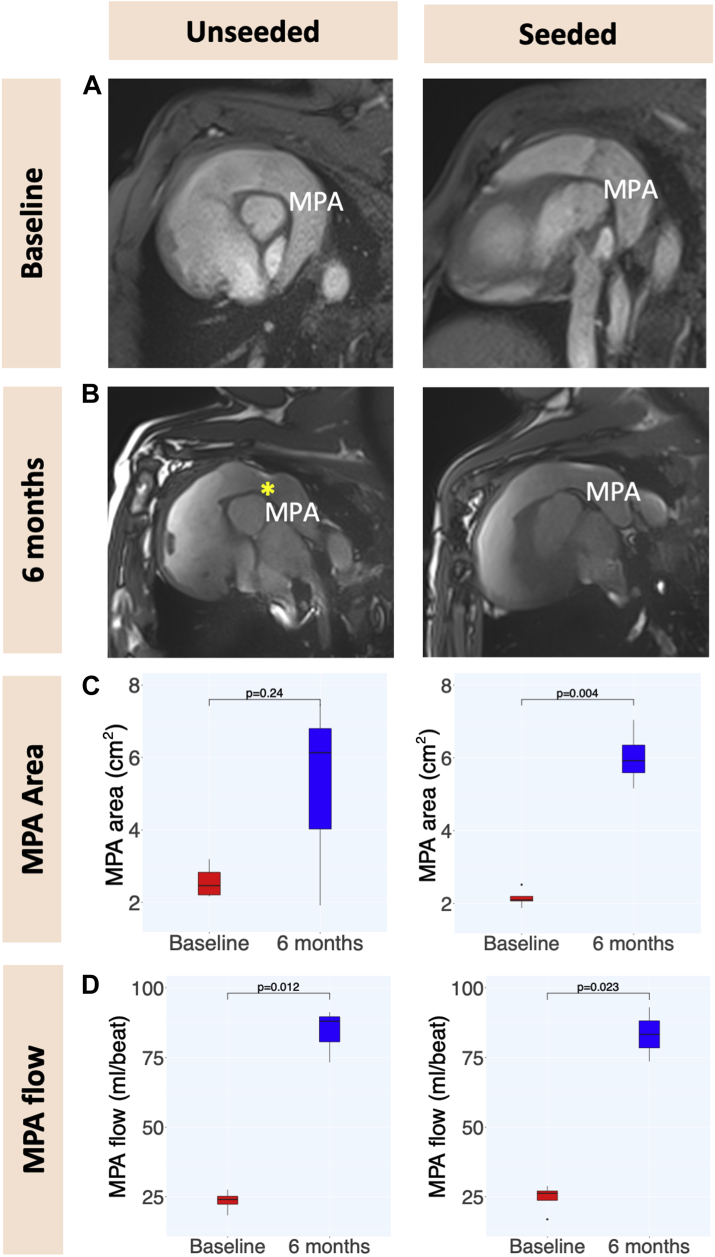
Cardiac Magnetic Resonance Analysis **(A)** Images of the RVOT/MPA at baseline and 6 months following graft implantation in seeded and unseeded animals. **(B)** Cardiac magnetic resonance long-axis view of the RVOT/MPA after implantation, showing lack of growth in an unseeded animal **(yellow asterisk)**, and better growth and remodeling with no graft stenosis in a similar picture of a seeded graft. **(C,D)** Box plots summarizing MPA area and flow measurements on cardiovascular magnetic resonance at baseline and at 6-month follow-up, in unseeded and seeded animals. MPA area growth was significant in seeded graft, but not in unseeded grafts (n = 4, ± SD). Abbreviations as in Figure 2.

The heart was accessed by median sternotomy, and cardiopulmonary bypass was established by cannulating the ascending aorta, the superior vena cava directly, and the inferior vena cava via the right atrial appendage. On the beating heart, an 8-mm segment of the MPA was excised, leaving the native pulmonary valve and annulus intact. The previously prepared circumferential conduit was then implanted as an interposition graft on the MPA, distal to the pulmonary valve (Figure 1B). Animals were extubated on the day of surgery and received intensive care for 24 hours postoperatively. Pain was managed with opioids and nonsteroidal anti-inflammatory drugs; noninvasive blood pressure, heart rate, oxygen saturation, temperature, and chest drain output were checked every 2 to 4 hours during this time. All animals underwent TTE (Figures 2A and 2B) and cardiac magnetic resonance (CMR) (Figures 3A and 3B) immediately before surgery and 6 months postoperatively. Peak velocities across the MPA and the RVOT were measured by TTE; MPA diameters (laterolateral and superoinferior) and MPA cross-sectional areas were measured by CMR. Animals were sacrificed with an intravenous injection of 150 mg/kg of pentobarbital sodium after completing the 6-month follow-up.

### Histology

Explanted samples were washed in phosphate-buffered saline and fixed overnight with 4% paraformaldehyde at 4°C. Fixed tissues were processed in a Thermo Excelsior AS (Thermo Fisher Scientific) and embedded with a Thermo HistoStar (Thermo Fisher Scientific) machine. Five-micrometer–thick sections were cut using a Shandon Finesse 325 microtome (Thermo Fisher Scientific). Slides were stored at 37^o^C overnight to dry completely before staining. Hematoxylin and eosin and Van Gieson’s stainings were performed using a Shandon Varistain 24-4 (Thermo Fisher Scientific) automated machine. Von Kossa staining was performed using a silver plating kit (In Vitro Diagnostic Medical Device) for the detection of microcalcification. Collagen and elastin contents of the explants in the Van Gieson’s staining were quantified using ImageJ software. Results were expressed as proportion of area occupied by collagen or elastin within the graft tissue.

### Immunohistochemistry

Paraffin-embedded sections were deparaffinized by 2 changes of clarene and rehydrated through an alcohol gradient. A heated antigen retrieval with 10 mM citrate buffer pH 6.0 was performed. Samples were blocked with 10% goat serum (Sigma-Aldrich) in phosphate-buffered saline for 30 minutes at room temperature and incubated with the unconjugated primary antibodies (α–smooth muscle actin [SMA], 1:100, Sigma-Aldrich; Isolectin B4-Biotin 1:100, Life Technologies; Elastin, 1:100, Santa Cruz Biotechnology) overnight at 4°C. Fluorophore-conjugated (Alexa Fluor 488 and Alexa Fluor 546, 1:400, Life Technologies) or chromogen-conjugated (horseradish peroxidase, 1:1,000, R &D Systems) secondary antibodies were incubated on the sections for 1 hour at room temperature in the dark. Incubation with 3,3’diaminobenzidine substrate (Abcam) was used for detection of horseradish peroxidase–derived signal. Nuclei were counterstained with 4′,6-diamidino-2-phenylindole (1:1000, Sigma-Aldrich) for fluorescent staining, or with hematoxylin for immunohistochemical staining. Slides were mounted with Hardset mounting medium (Vectashield). Images were taken with a Zeiss Observer.Z1 fluorescent microscope. ImageJ software was used to quantify the SMA and elastin expression in the tissue sections.

### Fluorescent in situ hybridization

Paraffin-embedded tissues were deparaffinized, rehydrated, and allowed to air-dry. Slides were incubated with 30% sodium bisulfite (Sigma-Aldrich) for 20 minutes at 37°C and washed with 2 × saline-sodium citrate (Thermo Fisher Scientific). Samples were incubated with a proteinase K (Qiagen) solution for 15 minutes at 45°C and then washed with 2 × saline-sodium citrate. Slides were rehydrated through an alcohol gradient and allowed to air-dry. The Y-chromosome probe mixture (Chrombios) was added to the samples. The slides were placed on a hotplate for 10 minutes at 80°C and then transferred overnight to a humidified chamber at 37°C. At the end of the hybridization, the samples were washed with 0.4 × saline-sodium citrate at 73°C and with 2 × saline-sodium citrate at room temperature. Nuclei were counterstained with 4′,6-diamidino-2-phenylindole (1:1,000) and mounted with Vectashield Hardset Mounting Medium. Fluorescent in situ hybridization (FISH) was performed on the seeded grafts before in vivo implantation and on seeded grafts explanted after 1 week and 2 months. The right ventricle of a male pig sacrificed at the time of umbilical cord collection was used as a positive control.

### Statistical analysis

Continuous variables are expressed as mean ± SD and were compared using unpaired or paired Student's *t*-test as appropriate. All tests were 2-sided with a level set at 0.05 for statistical significance. Data were recorded and subsequently tabulated with Microsoft Excel (VR Microsoft Corp). Statistical analyses were conducted using RStudio version 1.2.5042 (RStudio: Integrated Development for R. RStudio, Inc).

### Endpoints

The target follow-up for 9 of the operated animals was 6 months; these animals constituted the main study population. Animals receiving seeded and unseeded grafts were compared in terms of weight gain, histological features of the MPA, Doppler velocities across the RVOT/MPA, and CMR measurements of MPA diameters, area, and flow.

Furthermore, 2 animals were terminated 1 week and 2 months postoperatively, respectively, in order to quantify WJ-MSC population on the graft after surgery and to conduct a biodistribution study.

## Results

### In vivo assessment of WJ-MSCs engineered conduit implantation

The WJ-MSC-engineered grafts, shaped as conduits as described before, were used to replace the MPA of female piglets.^19^

Baseline animal weight was similar between groups (22.17 ± 2.75 kg for the unseeded group vs 19.25 ± 4.09 kg for the seeded group; *P =* 0.36). All operated animals survived the surgical procedure and had similar body weight gain at the end of follow-up (121.7 ± 25.65 kg for the unseeded group and 112.2 ± 29.8 kg for the seeded group; *P =* 0.67). One animal in each group died before the completion of the planned follow-up. The unseeded animal had completed the 6-month follow-up uneventfully and died at induction of general anesthesia just before undergoing the final CMR scan. The seeded animal had been in poor general condition since surgery and underwent planned termination 3 months postoperatively. In both cases, graft implantation was not thought to be related to the death after postmortem examination.

TTE showed no significant difference in peak velocity across the MPA graft at 6 months postoperatively compared to baseline, both in the seeded (1.59 ± 0.52 vs 1.10 ± 0.23 m/s, respectively; *P =* 0.13) and unseeded group (1.42 ± 0.03 vs 1.16 ± 0.21 m/s, respectively; *P =* 0.14) (Figure 2C). Similarly, there were no significant differences in terms of peak RVOT velocity at 6 months compared to baseline in the seeded group (1.22 ± 0.54 vs 0.82 ± 0.17 m/s, respectively; *P =* 0.16); however, peak RVOT velocity increased significantly at follow-up in the unseeded group (1.44 ± 0.18 vs 0.74 ± 0.19; *P =* 0.050) (Figure 2D).

CMR showed that laterolateral and superoinferior MPA diameters increased significantly at follow-up in the seeded group, whereas no significant growth was observed in the unseeded group. As a consequence, MPA area increased significantly at 6 months compared to baseline in the seeded group (6.01 ± 0.79 vs 2.15 ± 0.23 cm^2^; *P =* 0.004) (Figure 3C). On the other hand, MPA area did not change significantly in the unseeded group (5.17 ± 2.89 vs 2.57 ± 0.48 cm^2^; *P =* 0.24) (Figure 3C). Regarding MPA flow, both groups showed a significant increase at follow-up compared to baseline (81.15 ± 9.59 vs 23.50 ± 3.82 ml/beat; *P =* 0.012 in the unseeded group; and 83.27 ± 13.76 vs 24.58 ± 5.25 ml/beat; *P =* 0.023 in the seeded group) (Figure 3D).

### Histological ex vivo analysis of explanted tissues

**Figure 4:**
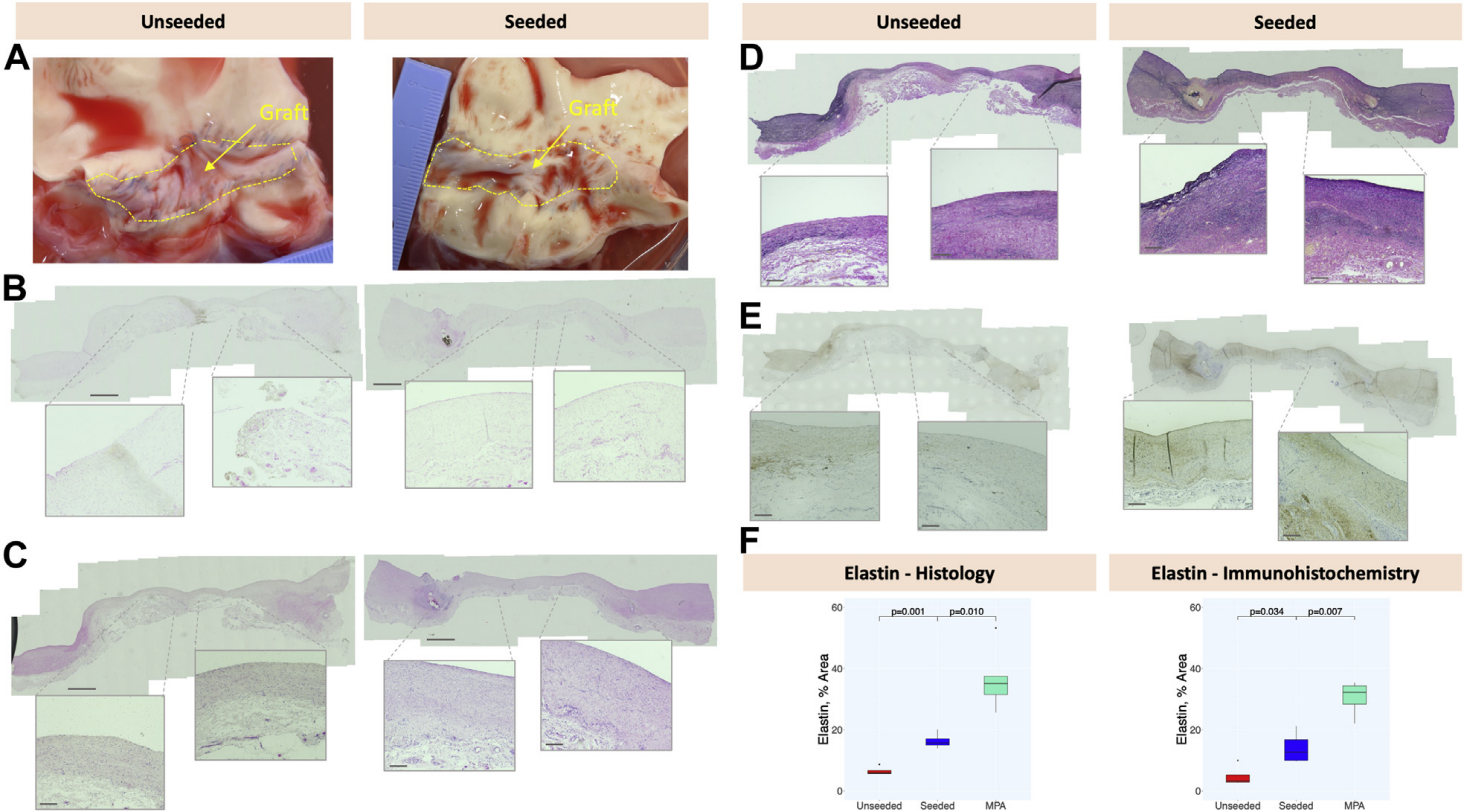
Histological Findings on Explanted MPAs and Grafts **(A)** Representative image of the explanted acellular and cell-engineered conduit. **(B)** Von Kossa staining showing no calcification in the grafts. **(C)** Hematoxylin and eosin staining showing extensive nucleation throughout the structure of seeded and unseeded grafts. **(D)** Van Gieson staining of collagen **(pink)** and elastin **(purple)** content of the grafts. **(E)** Immunohistochemistry showing elastin expression **(brown staining)** in the unseeded and seeded grafts. **(F)** Box plots showing elastin concentration, as quantified by histology and immunohistochemistry, in unseeded/seeded grafts and in the native MPA (n = 4, ± SD). Zoomed pictures in **B, C, D and E** indicated by **dashed lines.** Scale bars = 1,000 μm, 50 μm higher magnification. Abbreviation as in Figure 2.

At macroscopic inspection (Figure 4A) and on Von Kossa staining (Figure 4B), explanted grafts presented a smooth luminal surface with no signs of obstruction or tissue calcification and degradation in both groups.

Although hematoxylin and eosin images showed extensive nucleation throughout both seeded and unseeded grafts, a remarkably more organized cellular structure was detected in the seeded group (Figure 4C). Histological analysis showed a significantly higher content of elastin in the seeded group compared to the unseeded (16.36 ± 2.68 vs 6.5 ± 1.45 area percentage, respectively; *P =* 0.001), with this data being confirmed by immunohistochemical staining (14.06 ± 5.42 vs 4.8 ± 3.45 area percentage, respectively; *P =* 0.034), whereas no difference in collagen content was detected between the 2 groups (39.8 ± 4.47 vs 47.3 ± 12.68 area percentage, respectively; *P =* 0.33) as shown in Figures 4D to 4F.

**Figure 5:**
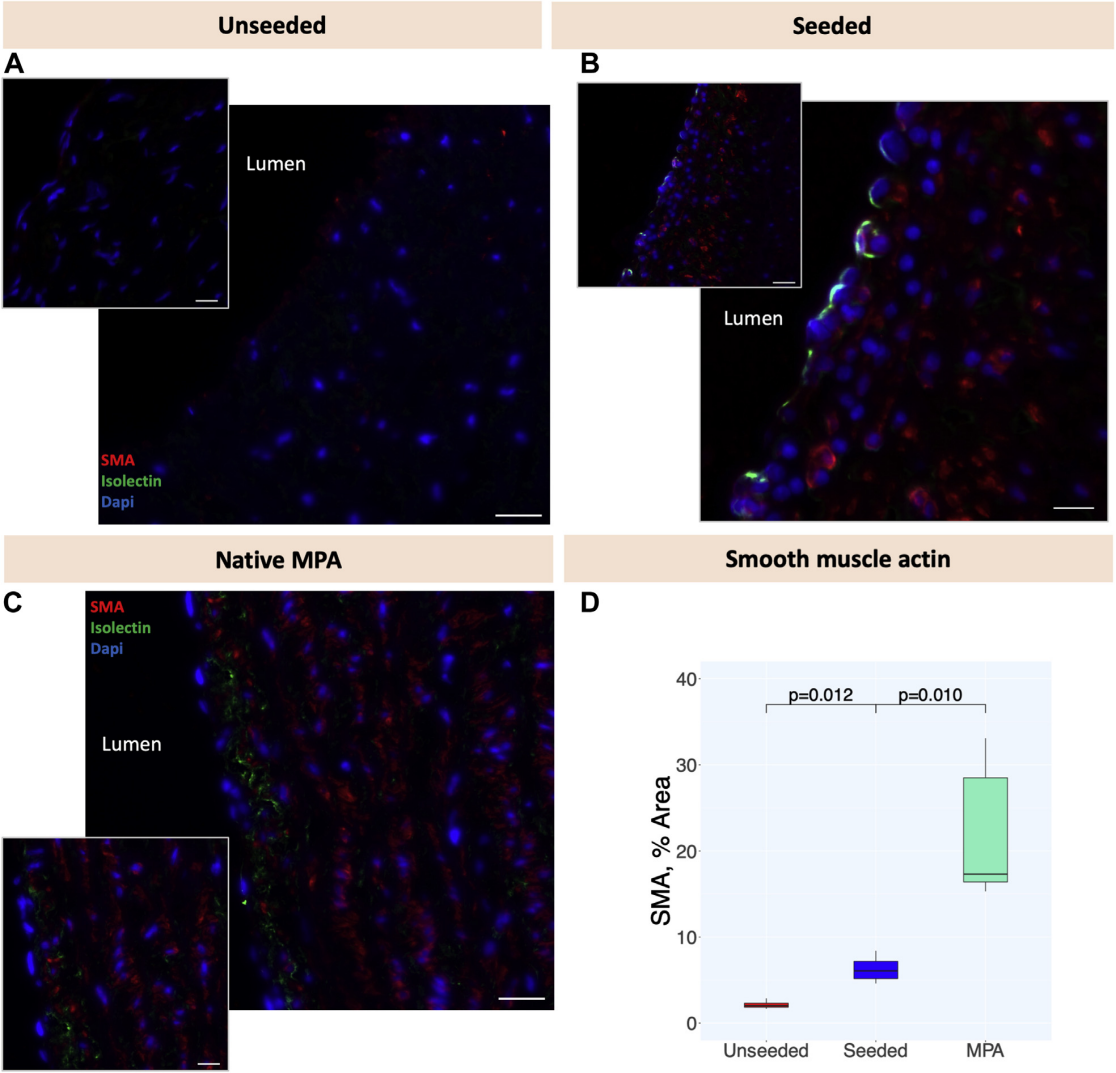
Immunohistochemistry of Explanted Native MPAs and Grafts Representative images showing longitudinal sections of the grafts, far from the anastomotic sites. A highly organized layer of smooth muscle cells (smooth muscle actin [SMA]–positive, **red**) and a newly formed layer of endothelial cells (isolectin-positive, **green**), similar to the native MPA **(C),** were more pronounced in the seeded graft **(B),** compared to the unseeded graft **(A).** 4′,6-diamidino-2-phenylindole was used to mark nuclei **(blue)**. The isolectin-positive cell concentration was similar between native MPA and seeded graft, whereas the slightly different staining pattern is most likely artifactual. Panoramic pictures **(small)** and corresponding zoomed pictures **(large)**. **(D)** Box plot showing the different SMA concentrations in unseeded/seeded grafts and in native MPA (n = 4, ± SD). Scale bars = 20 μm, 10 μm higher magnification. Abbreviation as in Figure 2.

As revealed by immunohistochemical staining, a newly formed layer of endothelial cells was detected on the luminal side. Moreover, a significantly higher concentration of SMA-stained cells repopulated the neotunica media of the seeded grafts compared to the acellular counterparts, (6.27 ± 1.68 vs 2.14 ± 0.53 area percentage, respectively; *P =* 0.012) (Figure 5).

The native MPA had a significantly higher level of SMA and elastin and significantly lower content of collagen compared to both graft types (Figures 4F and 5D).

**Figure 6:**
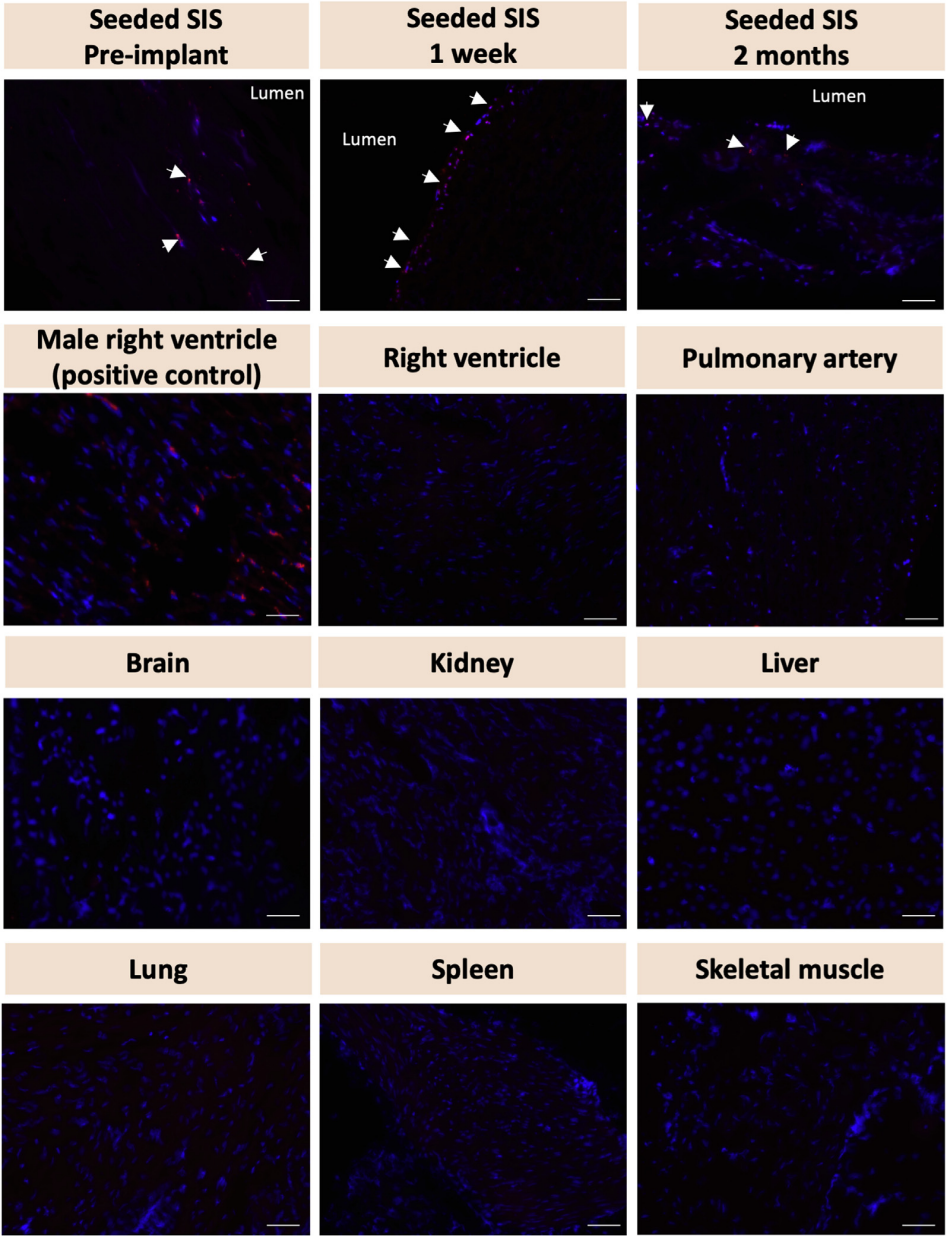
Y Chromosome Fluorescent In Situ Hybridization of the Explanted Grafts and Non-Target Tissues The male seeded cells (Y chromosome-positive, **red**) were detected on the MPA before implantation and 1 week and 2 months postoperatively. The presence of seeded cells decreased with time in vivo. No seeded cells were observed in the pulmonary artery away from the grafting site or in any of the non-target tissues after 2 months. The right ventricle of a male pig was used as a positive control. 4′,6-diamidino-2-phenylindole was used to mark nuclei **(blue)**. Scale bars = 50 μm. Abbreviation as in Figure 2.

We next examined the fate of implanted male donor cells into female recipients using FISH analysis of Y chromosome in the explanted grafts. Cells expressing the Y chromosome were detected on SIS before surgical implantation and in grafts explanted at 1 week postimplantation, whereas only few cells expressing the Y chromosome were observed at 2 months (Figure 6). Therefore, it is most likely that the smooth muscle cells and endothelial cells found in the explanted grafts at 6 months derive from the recipient rather than from donor WJ-MSCs. The decline in the number of implanted cells on the site of engraftment prompted us to further investigate whether the donor WJ-MSCs migrate from the graft to other tissues. The results from the biodistribution study showed that no male WJ-MScs were present in any of the analyzed tissues, thus excluding any potential cell migration and homing to non-target organs, typically observed in intravenously administered stem cells.

## Discussion

Although in vitro engineering of vascular grafts for heart defect correction has made enormous progress over the last decade, in vivo studies that prove their efficacy in suitable animal models that closely resemble the clinical scenario are often lacking.^20^, ^21^, ^22^, ^23^, ^24^, ^25^

Currently available biomaterials for valvar and vascular replacement in cardiac surgery do not rely on active integration with the host, but purely on mechanical properties and supposed lack of activation of the host’s immune reaction. Despite huge initial enthusiasm, commercially available porcine SIS (ProxiCor) has not shown any significant advantages over previous biomaterials from this perspective. The innovative step of our approach is to use cell-seeding technologies to develop a biomaterial which can not only be passively compatible with the host, but also actively promote the host’s regeneration.

This study is part of a stepwise process whose ultimate goal will be to develop a growing valved graft for the repair of conditions requiring surgical implantation of a right ventricle to pulmonary artery conduit in pediatric patients, such as tetralogy of Fallot with pulmonary stenosis or pulmonary atresia, and truncus arteriosus.

This work focused on the vascular component of such a graft. Traditionally, tissue-engineered valved conduits have been studied using the same biomaterial for both the vascular and the valvar portion of the graft, and they have been analyzed as a functional unit. Porcine SIS (ProxiCor), which is being used in various clinical applications of cardiovascular surgery with no evidence of immune rejection, has also been utilized in different animal models of valved conduit reconstruction, with ambiguous results.^23^, ^24^, ^25^, ^26^, ^27^ However, the biology and the biomechanics of the vascular wall significantly differ from those of the valvar leaflets; therefore, we believe that in a preclinical setting the 2 components must be addressed and analyzed separately as a first step.

In a proof-of-concept study, we have previously shown the feasibility of using a mesenchymal stem cell (MSC)–seeded SIS tissue-engineered graft for MPA and RVOT reconstruction in healthy piglets.^12^^,^^19^ Additionally, we upgraded our in vitro manufacturing process of this graft to clinical grade, Good Manufacturing Practice–compliant standards.^19^ Here, we assessed its function with a randomized controlled preclinical trial. Our study has 4 main findings.

First, when seeded with WJ-MSC and using our proposed technique, SIS is a reliable material for surgical reconstruction of the MPA, and it manifests potential for growth. According to our histological analysis, although both seeded and unseeded grafts revealed the absence of calcification, the seeded group exhibited a substantially higher host cell invasion and matrix protein concentration. In particular, the WJ-MSC–engineered conduit showed a significantly increased concentration of elastin in the neotunica media. In line with the latter finding, a significantly higher percentage of smooth muscle cells (SMA-stained), which are primarily involved in the synthesis and deposition of elastic fibers, repopulated the tunica media of the seeded graft compared to the acellular counterpart.^28^ The observation of newly synthetized elastic fibers is a notable result, given that the lack of mature elastin structures constitutes a serious limitation of tissue-engineering functional blood vessels, and it has been identified as a flaw of nonseeded SIS vascular grafts.^28^^,^^29^

Second, the presence of donor WJ-MSCs in the seeded graft, during the first few postoperative weeks, is crucial for the observed growth, remodeling, and a neo-endothelialization of the luminal side of the seeded conduits. Because FISH analysis showed a decrease in the number of WJ-MSC in the graft area within 2 months postoperatively, the most likely mechanism explaining our findings is paracrine stimulation of endogenous cell recruitment and migration exerted by the implanted donor WJ-MSCs. This hypothesis is also supported by works previously published by ours and other groups.^11^^,^^12^^,^^15^ Sugiura et al^15^ showed that induced pluripotent stem cell–derived cardiomyocytes seeded onto a biodegradable cardiac patch, implanted in an RVOT rat model, were no longer present after 4 weeks. However, Sugiura et al^15^ found that α-actinin positive cells were significantly greater in the seeded group than in the unseeded group, and surmised that seeded induced pluripotent stem cell derived cardiomyocytes might influence the regeneration of host cardiomyocytes via a paracrine mechanism.

The fate of the donor cells in the host body is currently not clear, as no trace of the implanted cells was found in non-target organs. Theoretically, efferocytosis (phagocytosis of donor MSCs and release of chemokines and growth factors by tissue-resident macrophages) may have contributed to MSC clearance, tissue regeneration, and immunomodulation, although direct evidence of efferocytosis was not sought in this work.^30^, ^31^, ^32^ Overall, further elucidation regarding the fate of WJ-MSCs and their long-term survival is warranted. However, donor cells’ number decrease and potential disappearance over time could be beneficial as it would reduce immune reaction when allogeneic donor cells are used.

Third, seeding with allogeneic cells was both effective (as demonstrated by the above findings) and safe (as shown by our biodistribution data revealing no cell migration to non-target organs). This is consistent with previous reports showing that seeding and engraftment on a solid scaffold has the advantage of overcoming the limitation of cell migration and homing to non-target organs, typically observed after stem cell intravenous transfusion.^33^^,^^34^

Despite the obvious appeal of autologous cells, we believe that allogeneic cells have significant advantages in terms of production, storage, and availability on an urgent basis, which ultimately determine a higher translational potential. Additionally, the potential disappearance of the implanted donor cells over time would further support the safety of the allogeneic approach.

In this study we used an allogeneic porcine cellular product instead of testing the human cell construct to avoid the risk of cross-species immunogenic response and the side effects of immunosuppressive therapy typical of xenogeneic cells.^35^ This decision was supported by the “Guideline on Human Cell-based Medicinal Products” from the Committee for Medicinal Product for Human Use which asserts that sufficiently characterized analogous animal-derived cells may be used for allogeneic cell-based products destined to preclinical trials.^35^ The surrogate cell product we have developed for this study had been extensively validated through characterization of the cell phenotype, function, and capacity to integrate and give rise to a living tissue.^19^

Fourth, our histologic findings were supported by in vivo assessment through clinically relevant imaging modalities, which could ultimately increase the translational impact of this study. The seeded grafts showed significant increase in cross-sectional area on CMR over the study period, whereas no significant change was observed in unseeded grafts.

Regarding Doppler, the interpretation of the increase in peak RVOT velocity that we observed in the unseeded group is not immediate; it could be hypothesized that smaller unseeded MPAs triggered a degree of RVOT hypertrophy that can account for the higher velocities, but we did not analyze RVOT hypertrophy in this study. However, although significantly higher than at baseline, the RVOT velocities in unseeded animals at follow-up were in the range of clinical normality; therefore, the statistical significance potentially does not translate into a clinically relevant finding.

Overall, our findings differ from most published work in the scientific literature. Multiple investigators have reported failures with SIS over the past 10 years, both in preclinical and clinical scenarios.^25^, ^26^^,^^36^, ^37^, ^38^, ^39^, ^40^ However, several papers have focused on this biomaterial used as a valve leaflet patch and/or in high pressure districts^37^^,^^40^; moreover, histologic changes in preclinical studies were mostly assessed on nonseeded grafts.^23^, ^24^, ^25^^,^^27^ It could be argued that seeded porcine SIS might not exhibit the same behavior observed in this work when implanted in humans as a result of cross-species immune reaction to the graft. However, histological analyses of explanted SIS from human show a histologic pattern of chronic inflammation that is similar to that observed in SIS explanted from pigs.^27^^,^^41^

To our knowledge, our work is the first combining several aspects that we believe to be essential to our research goal: it is focused exclusively on vascular reconstruction, it compares in a randomized trial seeded to unseeded grafts, and it assesses growth. In our opinion, this specific design can account for the discrepancy between failures reported by other groups and our positive findings. Furthermore, this is one of the first studies in which CMR has been used to assess implanted grafts on a large animal model.

### Study limitations

Despite its randomized design, our study’s main limitation is that the number of animals was relatively small. Therefore, normality could not be tested for continuous variables. Furthermore, type I error adjustment for multiple comparisons was not done, so results should be interpreted with caution. Nevertheless, this work focused mainly on the translational potential of our seeded grafts: although deeper understanding of the mechanisms behind MSC reduction over time is warranted, it is beyond the scope of this study.

## Conclusions

SIS seeded with WJ-MSCs is a reliable biomaterial for MPA reconstruction in a large animal model that exhibits growth potential. The superiority of our construct to unseeded SIS was demonstrated by both histological and CMR imaging findings.

Perspectives **COMPETENCY IN MEDICAL KNOWLEDGE:** Integration of grafted stem cells into the host target tissue while avoiding non-target organs is a key prerequisite of tissue-engineered grafts. Allogeneic MSC derived from Wharton’s Jelly, when implanted onto porcine small intestinal submucosa, integrate into the swine main pulmonary artery and promote graft repopulation by host cells to form an organized vascular structure with growth potential.

**TRANSLATIONAL OUTLOOK:** Our work shows for the first time in a randomized animal model study the importance of repopulating the SIS with allogeneic stem cells for effective reconstruction of the main pulmonary artery. The translation of our tissue-engineering approach into a clinical and efficacy study might pave the way to new modalities of intervention for effective surgical restoration of pulmonary artery and RVOT function in tetralogy of Fallot and truncus arteriosus patients.

## Funding Support and Author Disclosures

This study was supported by grants from the Sir Jules Thorn Charitable Trust, the Enid Linder Foundation, and the British Heart Foundation. The authors have reported that they have no relationships relevant to the contents of this paper to disclose.
